# *ACTB* Methylation in Blood as a Potential Marker for the Pre-clinical Detection of Stroke: A Prospective Nested Case-Control Study

**DOI:** 10.3389/fnins.2021.644943

**Published:** 2021-05-14

**Authors:** Chunlan Liu, Qiming Yin, Mengxia Li, Yao Fan, Chong Shen, Rongxi Yang

**Affiliations:** ^1^Department of Epidemiology, School of Public Health, Nanjing Medical University, Nanjing, China; ^2^Division of Clinical Epidemiology, Affiliated Geriatric Hospital of Nanjing Medical University, Nanjing, China

**Keywords:** DNA methylation, stroke, *ACTB* gene, pre-clinical detection, marker

## Abstract

**Background:**

Stroke is the second leading cause of death worldwide. If risk of stroke could be evaluated early or even at a preclinical stage, the mortality rate could be reduced dramatically. However, the identified genetic factors only account for 5–10% of the risk of stroke. Studies on the risk factors of stroke are urgently needed. We investigated the correlation between blood-based β-actin (*ACTB*) methylation and the risk of stroke in a prospective nested case-control study.

**Methods:**

The methylation level of *ACTB* was quantitatively determined by mass spectrometry in 139 stroke cases who developed stroke within 2 years after recruitment and 147 age- and sex-matched controls who remained stroke-free in a median follow-up of 2.71 years.

**Results:**

We observed a highly significant correlation between hypomethylation of one CpG site of *ACTB* and increased risk of stroke in an onset-time-dependent manner (for onset time ≤ 1.5 years: odds ratio (OR) per + 10% methylation = 0.76, *P* = 0.001; for onset time ≤ 1.32 years: OR per + 10% methylation = 0.59, *P* = 7.82 × 10^–7^; for onset time ≤ 1 year: OR per + 10% methylation = 0.43, *P* = 3.00 × 10^–6^), and the increased cumulative incidence of stroke (log-rank *P* = 3.13 × 10^–7^). Neighboring CpG sites showed an inverse correlation with age and drinking status in controls (*P* < 0.05) but not in stroke cases.

**Conclusion:**

We firstly reported the blood-based *ACTB* methylation as a marker for the risk evaluation and preclinical detection of stroke, which can be further modified by age and drinking.

## Introduction

Stroke represents the second leading cause of death worldwide (GBD 2016 Causes of Death Collaborators, 2017) and has contributed to almost 5% of all disability-adjusted life-years (Feigin et al., 2017). In 2016, the worldwide lifetime risk of stroke for people aged 25 years and above approaches 25%; in China, the risk is estimated to be as high as 39.3% (41.1% in men and 36.7% in women) (GBD 2016 Lifetime Risk of Stroke Collaborators et al., 2018).

Stroke is a multifactorial disease and is related to several genetic factors, and genetic-environmental interaction (Benjamin et al., 2017). β-actin (encoded by *ACTB*), a highly conserved cytoskeletal protein, is widely distributed in all eukaryotic cells (Rubenstein, 1990). β-actin is characterized by its ability to polymerize and participate in a variety of cell functions, such as maintenance of cell shape, cell migration, division, growth, and signal transduction (Herman, 1993; Chen et al., 2016). Moustafa-Bayoumi et al. (2007) suggested that elevated actin polymerization and stress fiber formation would generate mechanical force to trigger the hypertrophic signaling pathway, subsequently resulting in vascular remodeling and hypertension that can reduce blood flow in brain and alter the mechanics and function of cerebral blood vessels, and ultimately increase the risk of stroke (Legrand et al., 1993; Ibrahim et al., 2006). Our previous study showed that *ACTB* polymorphisms may contribute to the genetic susceptibility to stroke (Yang et al., 2020), although the mechanism of *ACTB* polymorphisms to stroke remains unclear. Taken together, all the identified stroke-related genetic factors account for only 5–10% of the risk of stroke (Bevan et al., 2012; Malik and Dichgans, 2018). Studies on stroke risk factors are still urgently needed.

Epigenetic factors may contribute new hints for the understanding and evaluation of the risk of stroke (Felling and Song, 2015). Epigenetics refers to DNA modifications affecting gene expression that are not based on mutation of the underlying DNA sequence (Nicoglou and Merlin, 2017). DNA methylation, a major type of epigenetic regulation, mainly occurs at the cytosine of a cytosine-phosphate-guanine (CpG) dinucleotide in differentiated mammalian cells (Bird, 2007). Candidate approach studies have found certain stroke-associated aberrant DNA methylation patterns, such as altered methylation in *LINE-1*, *ABCB1*, and *CBS* genes in peripheral blood, but mainly in case-control studies with small sample sizes (Lin et al., 2014; Yang et al., 2015; Wang et al., 2019). So far, no data are available about the association between blood-based *ACTB* methylation and stroke, especially in prospective studies.

This study aimed to explore the relationship between DNA methylation of the *ACTB* gene in peripheral blood and stroke risk in a nested case-control study from a prospective cohort with a total of 11,151 subjects. The blood samples were collected at the time point of enrollment when all individuals were reported to be stroke-free. The subjects who later developed stroke within 2 years after enrollment in the cohort were defined as cases, and those who remained stroke-free during a median follow-up of 2.71 years were selected as controls matched by age and sex.

## Materials and Methods

### Study Population of Prospective Cohort

This study was approved by the ethics committee of Nanjing Medical University. Written informed consent for participation in the study was obtained from all participants.

Subjects for this nested case-control study were selected a prospective cohort from Jurong City, Jiangsu Province. This prospective cohort study was conducted from October to November 2015, and a total of 11,151 subjects aged ≥18 years were recruited. All individuals were reported stroke-free when recruited. For all the subjects, demographic data including age, sex, nationality, status of smoking, alcohol consumption frequency, and history of hypertension and diabetes were recorded through questionnaires, along with anthropometric measurements including weight, height, and blood pressure at baseline. Smoking status was divided into three groups: non-smokers, individuals who had ever smoked ≤100 cigarettes during a lifetime; former smokers, individuals who had stopped smoking for ≥1 year prior to the study; current smokers, individuals who were smoking within 1 year (Hughes et al., 2004). Drinking status was categorized into those who never drink (non-drinkers) and drinkers who currently or previously drink ≥2 times per week for at least 6 months per year (Millwood et al., 2013).

Peripheral blood samples were deposited in ethylenediamine tetraacetic acid tubes and kept at 4°C for up to 4 h before store at -80°C till usage. Additionally, peripheral venous blood was sampled to measure proportion of white blood cell types, the level of total cholesterol (TC), triglycerides (TG), high−density lipoprotein cholesterol (HDL−C), low−density lipoprotein cholesterol (LDL−C), and glucose (GLU).

Incidence of stroke was identified by the local hospitals, centers for disease control and community health service centers. All of the 139 subjects who developed stroke within 2 years after enrollment of the cohort were included in this study as cases. A total of 150 age and sex-matched individuals who remained stroke-free during the follow-up time were selected as controls. After excluding 3 low-quality clotted blood samples, a total of 147 controls were finally included in this study. The demographic and clinical characteristics of participants in this nested case-control study are shown in Table 1.

**TABLE 1:** Demographic and clinical characteristics of participants in the nested case-control study.

Characteristics	Controls	Stroke cases	t/χ ^2^	*P-*value
			
	(*n* = 147)	(*n* = 139)		
Age (year)	67.59 ± 9.11	67.64 ± 9.51	0.046	0.963
Sex				
Male	84 (57.1%)	81 (58.3%)	0.037	0.847
Female	63 (42.9%)	58 (41.7%)		
BMI (kg/m^2^)	24.75 ± 3.20	24.94 ± 3.52	0.467	0.641
SBP (mmHg)	147.43 ± 20.18	147.73 ± 22.28	0.119	0.906
DBP (mmHg)	81.34 ± 9.78	81.16 ± 11.86	0.138	0.890
Smoking status				
Current smokers	37 (25.2%)	35 (25.2%)	0.013	0.994
Former smokers	10 (6.8%)	9 (6.5%)		
Non-smokers	100 (68.0%)	95 (68.3%)		
Drinking status				
Yes	47 (32.0%)	47 (33.8%)	0.110	0.801
No	100 (68.0%)	92 (66.2%)		
History of hypertension				
Yes	88 (59.9%)	96 (69.1%)	2.636	0.110
No	59 (40.1%)	43 (30.9%)		
History of diabetes				
Yes	28 (19.0%)	27 (19.4%)	0.007	1.000
No	119 (81.0%)	112 (80.6%)		
TC (mmol/L)	5.11 ± 0.91	5.20 ± 0.93	0.882	0.379
TG (mmol/L)	1.57 ± 1.01	1.56 ± 0.88	0.039	0.969
HDL-C (mmol/L)	1.54 ± 0.46	1.55 ± 0.42	0.199	0.843
LDL-C (mmol/L)	2.88 ± 0.80	2.96 ± 0.75	0.886	0.377
Glucose (mmol/L)	6.58 ± 2.25	6.64 ± 2.24	0.234	0.815
Leukocytes (mil/mm^3^)	5.60 ± 1.51	5.99 ± 1.47	2.177	0.030
Neutrophils (%)	56.31 ± 7.95	58.50 ± 9.21	2.149	0.032
Lymphocytes (%)	35.67 ± 7.47	34.22 ± 8.71	1.502	0.134
Monocytes (%)	4.59 ± 1.18	4.44 ± 1.19	1.032	0.303

**BMI, body mass index; SBP, systolic blood pressure; DBP, diastolic blood pressure; TC, total cholesterol; TG, triglycerides; HDL-C, high-density lipoprotein cholesterol; LDL-C, low-density lipoprotein cholesterol.**

### DNA Extraction and Bisulfite Conversion

Genomic DNA was isolated using the Genomic DNA Extraction Kit as described previously (Yin et al., 2020). One microgram of genomic DNA was bisulfite converted by the EZ-DNA Methylation Gold kit (Zymo Research, Orange County, United States) according to the manufacturer’s instructions. After bisulfite treatment, all non-methylated cytosine (C) bases in CpG sites were converted to uracil (U), whereas all methylated C bases remained C. The samples from stroke cases and controls were processed in parallel.

### MALDI-TOF Mass Spectrometry

MALDI-TOF mass spectrometry (Agena Bioscience, San Diego, CA, United States) was used to determine the levels of DNA methylation quantitatively as described previously (Yang et al., 2017). The bisulfate- converted DNA was amplified by bisulfite-specific primers. The polymerase chain reaction (PCR) primer pairs of *ACTB* were as follow: forward primer: aggaagagagGGGATTTGATTGATTATTTTATGAAGA, reverse primer: cagtaatacgactcactatagggagaaggctACCACAAAACTCCATACCTAAAAAA. Uppercase letters indicate the sequence-specific primer regions, and lowercase letters indicate non-specific tags. The sequence of the PCR amplicon is provided in Supplementary Figure 1. This amplicon, located at the CpG island shore where differential methylation occurs frequently (Doi et al., 2009; Irizarry et al., 2009; Feber et al., 2011), covers the translation region of exon 4 of the *ACTB* gene and part of the fourth intron. There are no SNPs located at the primer regions or overlapped with the CpG sites. The PCR products were processed according to the manufacturer’s instructions of Agena EpiTyper Assay, further cleaned by resin, and then distributed into a 384 SpectroCHIP by a Nanodispenser. For each batch of MassARRAY analysis, case-control pairs were randomly arranged, and samples from stroke cases and controls were treated and analyzed in parallel in all the processes. The methylation intensities of the CpG sites in the nearby CpG island are mostly less than 0.05 (data not published, available upon request), which is lower than the accuracy limitation of the MassArray, and thus were not investigated in this study.

### Quantitative Real-Time PCR

Total RNA was isolated from peripheral blood leucocytes of each sample, then reverse transcribed into cDNA using the PrimeScript^TM^ RT Reagent Kit (Takara, RR047A, Japan). Quantitative real-time PCR was performed for the *ACTB* gene and the housekeeping gene glyceraldehyde-3-phosphate dehydrogenase (*GAPDH*) as an endogenous control using 2 × SYBR Green qPCR Master Mix (Bimake, B21202, Houston, TX, United States). The relative expression of *ACTB* for each sample was calculated according to the 2^–Δ^ ^Δ^ ^*ct*^ method via normalization to *GAPDH*.

### Statistical Analysis

Quantitative variables with normal distribution were expressed as mean ± standard deviation (SD), and differences between cases and controls were assessed with unpaired Student’s *t*-tests. Quantitative variables with non-Gaussian distribution were expressed as median (interquartile range), and the differences between cases and controls were assessed with Mann-Whitney U tests. Qualitative variables were compared using the Chi square (χ^2^) test. Unconditional logistic regression was used to estimate the association between the level of *ACTB* methylation and stroke by calculating odds ratios (ORs) and the 95% confidence intervals (CIs) as well as to adjust for covariates. The correlations between *ACTB* methylation and onset time of stroke in cases, between *ACTB* methylation and age, and between *ACTB* methylation and drinking in controls and in cases were all assessed by Spearman’s rank correlation coefficients. All statistical analyses were performed in SPSS version 25.0 (SPSS Inc., Chicago, United States) and the software R version 3.6.0. A two−tailed *P* < 0.05 was considered statistically significant.

## Results

### Demographic and Clinical Characteristics of the Participants

Demographic and clinical characteristics of individuals in the nested case−control study are shown in Table 1. Stroke cases showed slightly higher but significant leukocyte counts (5.99 vs. 5.60, *P* = 0.030) and neutrophil proportion (58.50 vs. 56.31%, *P* = 0.032) than controls. Between stroke cases and controls, there were no significant differences in age, sex, BMI, SBP, and DBP, smoking status, drinking status, history of hypertension, diabetes, TC, TG, HDL-C, and LDL−C, glucose and proportions of lymphocyte and monocyte. Median follow-up time (time between blood draw and analysis cutoff date) of controls was 2.71 years. Median onset time (time between blood draw and initial diagnosis of stroke) of stroke cases was 1.32 years.

### Onset-Time-Dependent Association Between *ACTB* Methylation and Stroke

**FIGURE 1:**
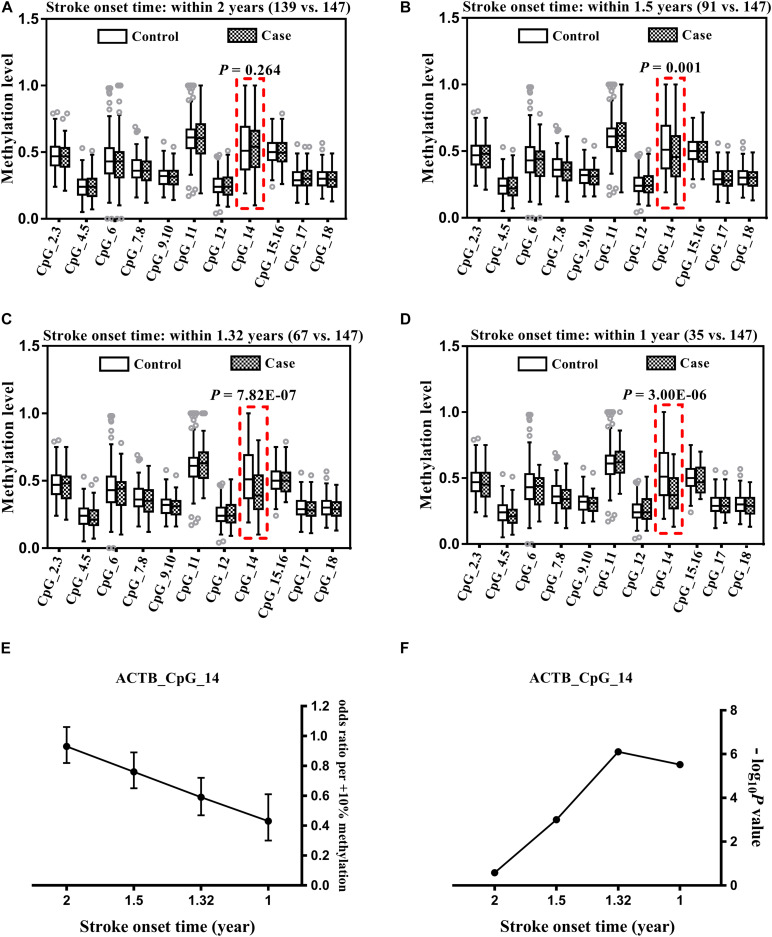
Association between *ACTB* methylation in peripheral blood and stroke. The analysis was performed for stroke cases with onset time < 2 years **(A)**, ≤1.5 years **(B)**, ≤1.32 years **(C)**, and ≤1 year **(D)**. The box plots show the distribution of *ACTB* methylation levels in stroke cases and controls. The *P*-values were calculated by logistic regression adjusting for BMI, smoking, drinking, hypertension, diabetes, TC, TG, HDL-C, LDL-C, leukocyte counts and proportions of neutrophil, lymphocyte and monocyte. The circles indicate outliers. **(E)** The OR per + 10% methylation of ACTB_CpG_14 in stroke cases with onset time <2, ≤1.5, ≤1.32, and ≤1 year were based on logistic regression analysis adjusting for BMI, smoking, drinking, hypertension, diabetes, TC, TG, HDL-C, LDL-C, leukocyte counts and proportions of neutrophil, lymphocyte and monocyte. **(F)** The *P*-values of association between ACTB_CpG_14 and stroke cases with onset time <2, ≤1.5, ≤1.32 and ≤1 year transformed by -log_1__0_*P*.

To evaluate the correlation between *ACTB* methylation and stroke in peripheral blood, an amplicon including 11 CpG sites was determined by Agena MALDI-TOF mass spectrometry. Comparing the 147 controls and all the 139 cases who developed stroke within 2 years, no significant associations between all the 11 CpG loci and stroke were observed even after adjusting for BMI, smoking, drinking, hypertension, diabetes, TC, TG, HDL-C, LDL-C, leukocyte counts and proportions of neutrophil, lymphocyte and monocyte (all *P-*values > 0.05, Figure 1A and Supplementary Table 1).

Since the onset time of stroke may influence the methylation levels of *ACTB*, we performed additional analyses comparing controls and stratified stroke cases by time of onset. Stroke cases with onset time ≤ 1.5 years showed significantly lower ACTB_CpG_14 methylation levels than controls [median = 0.46 (interquartile range, IQR = 0.31–0.61) and 0.51 (IQR = 0.37–0.69) for cases and controls, respectively, OR per + 10% methylation = 0.76, 95% CI: 0.65–0.89, *P* = 0.001 by logistic regression adjusting for BMI, smoking, drinking, hypertension, diabetes, TC, TG, HDL-C, LDL-C, leukocyte counts and proportions of neutrophil, lymphocyte and monocyte, Figure 1B and Supplementary Table 2]. Interestingly, the differences became more pronounced in stroke cases with onset time ≤ 1.32 years (OR per + 10% methylation = 0.59, 95% CI: 0.47–0.72, *P* = 7.82 × 10^–7^ by logistic regression adjusting for BMI, smoking, drinking, hypertension, diabetes, TC, TG, HDL-C, LDL-C, leukocyte counts and proportions of neutrophil, lymphocyte and monocyte, Figure 1C and Supplementary Table 3), and especially in cases with onset time ≤ 1 year (OR per + 10% methylation = 0.43, 95% CI: 0.30–0.61, *P* = 3.00 × 10^–6^ by logistic regression adjusting for BMI, smoking, drinking, hypertension, diabetes, TC, TG, HDL-C, LDL-C, leukocyte counts and proportions of neutrophil, lymphocyte and monocyte, Figure 1D and Supplementary Table 4). Overall, there was a clear trend of decreasing in OR per + 10% methylation of ACTB_CpG_14 and -log_1__0_*P*-value in stroke cases with onset time <2, ≤1.5, ≤1.32, and ≤1 year (Figures 1E,F).

None of the other 10 CpG sites in the *ACTB* amplicon showed an association with stroke at any time of onset (Figures 1A–D and Supplementary Tables 1–4).

### Correlation Between *ACTB* Methylation and Onset Time of Stroke

**FIGURE 2:**
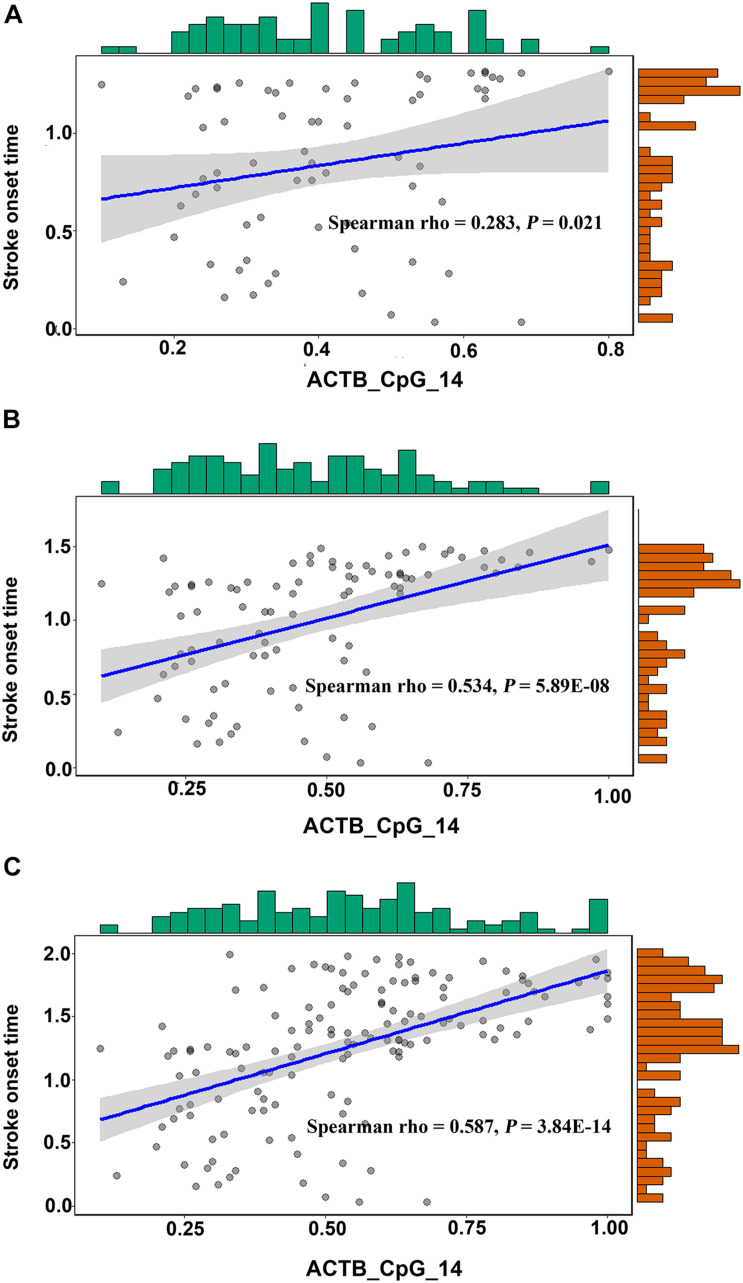
Positive correlation between ACTB_CpG_14 methylation and onset time of stroke for cases with onset time ≤1.32 years **(A)**, ≤1.5 years **(B)**, and <2 years **(C)**.

For stroke cases with time of onset ≤1.32, ≤1.5, and <2 years, the methylation levels of ACTB_CpG_14 showed increasingly positive and significant correlation with onset time of stroke (Spearman rho = 0.283, 0.534, and 0.587, respectively, *P* < 0.05 for all, Figure 2 and Supplementary Table 5). No correlation was observed between the methylation levels of ACTB_CpG_14 and time for stroke cases with onset ≤ 1 year, which might due to the very limited sample size of only 35 subjects (Supplementary Table 5).

The methylation levels of other 10 CpG sites in *ACTB* amplicon had no correlation with onset time of stoke (Supplementary Table 5).

### Hypomethylation of ACTB_CpG_14 Is Associated With Cumulative Incidence of Stroke

**FIGURE 3:**
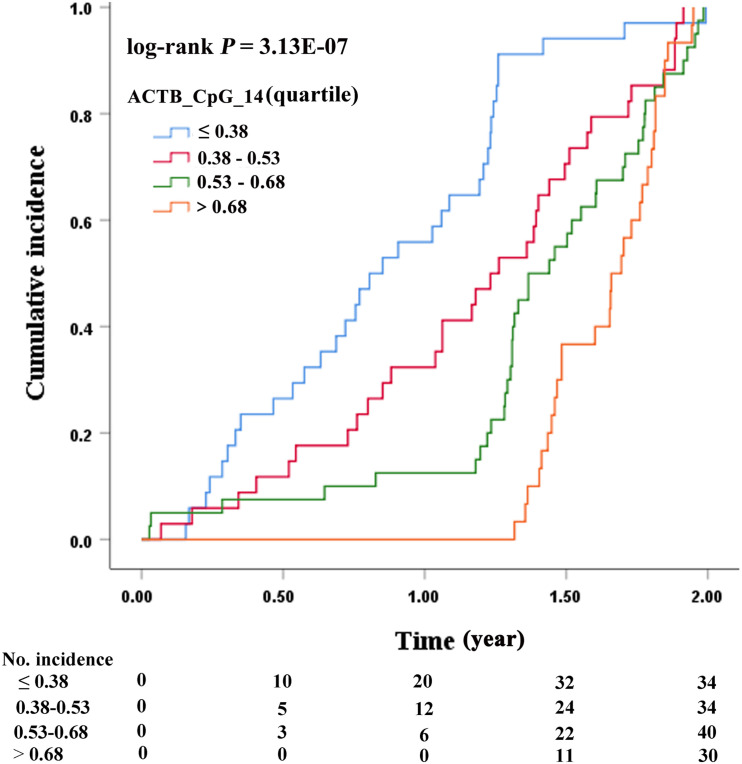
Association between ACTB_CpG_14 methylation and cumulative incidence of stroke by cumulative incidence curve.

Kaplan-Meier analysis was carried out to evaluate the association between ACTB_CpG_14 methylation levels and cumulative incidence of stroke. Figure 3 showed that compared to the quartile with the highest methylation level (Q4, methylation level > 68%), the three lower quartiles of ACTB_CpG_14 methylation levels (Q1–Q3) were associated with an increased cumulative incidence of stroke (log-rank *P* = 3.13 × 10^–7^) and earlier incidence of stroke.

### Association Between *ACTB* Methylation, Age, and Drinking

Previous studies suggested the interaction between DNA methylation and both age and lifestyle (Zykovich et al., 2014; Wilson et al., 2017; Liu et al., 2018; Zhang et al., 2018); we therefore tested the correlation between the methylation levels of *ACTB* and age, sex, and the status of smoking and drinking in all 147 controls and in all 139 cases who developed stroke within 2 years after enrollment.

**FIGURE 4:**
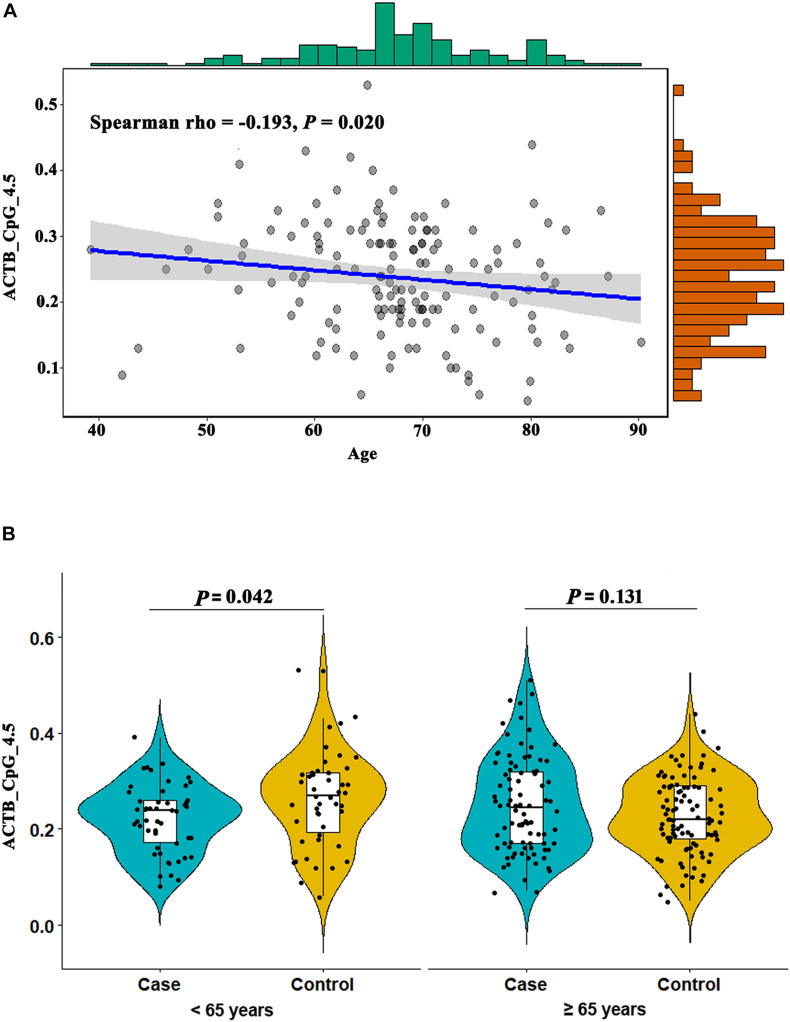
Association between ACTB_CpG_4.5 methylation and age. **(A)** The inverse correlation between ACTB_CpG_4.5 methylation and age in controls. **(B)** Association between ACTB_CpG_4.5 methylation in peripheral blood and stroke stratified by age (65 years). The box plots show the distribution of ACTB_CpG_4.5 methylation levels in stroke cases and controls. The black dots represent the individual data of ACTB_CpG_4.5 methylation levels. The *P*-values were calculated by logistic regression adjusting for BMI, smoking, drinking, hypertension, diabetes, TC, TG, HDL-C, LDL-C, leukocyte counts and proportions of neutrophil, lymphocyte and monocyte.

The methylation level of ACTB_CpG_4.5 was inversely correlated with age in controls (Spearman rho = -0.193, *P* = 0.020, Figure 4A), but not with age in stroke cases (Supplementary Table 6), which indicated that ACTB_CpG_4.5 methylation might be an age-dependent factor for the risk of stroke. Thus, we further examined the association between *ACTB* methylation and stroke stratified by 65 years, the age which is widely used for stroke risk stratification and stroke prevention in most guidelines (Chao et al., 2016). In subjects under the age of 65 years, ACTB_CpG_4.5 showed significantly lower methylation levels in cases than in controls [median = 0.24 (IQR = 0.17–0.27) and 0.27 (IQR = 0.19–0.32) for cases and controls, respectively, OR per + 10% methylation = 0.56, 95% CI: 0.33–0.98, *P* = 0.042 by logistic regression adjusting for BMI, smoking, drinking, hypertension, diabetes, TC, TG, HDL-C, LDL-C, leukocyte counts and proportions of neutrophil, lymphocyte and monocyte, Figure 4B and Supplementary Table 7]. However, this association was not observed in subjects above 65 years old (Figure 4B and Supplementary Table 7).

**FIGURE 5:**
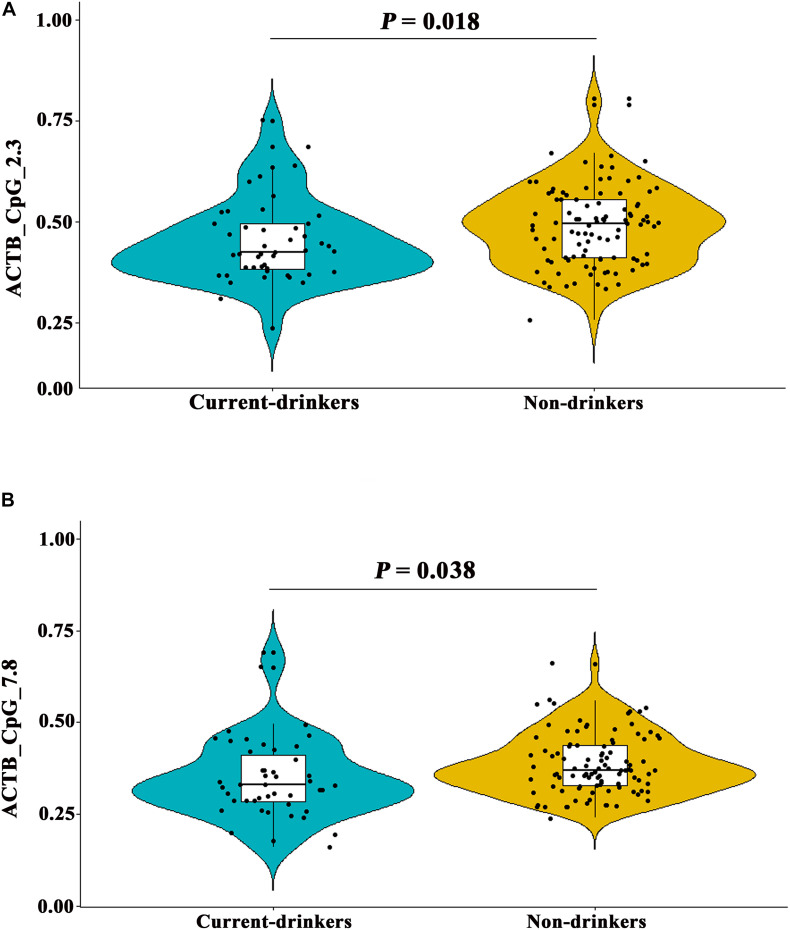
Distribution of ACTB_CpG_2.3 and ACTB_CpG_7.8 methylation between current-drinkers and non-drinkers. The box plots show the distribution of CpG_2.3 and CpG_7.8 methylation levels in current-drinkers and non-drinkers. The black dots represent the individual data of CpG_2.3 and CpG_7.8 methylation levels. The *P*-values were calculated by logistic regression adjusting for age and sex. **(A)** Difference of ACTB_CpG_2.3 methylation levels between current-drinkers and non-drinkers. **(B)** Difference of ACTB_CpG_7.8 methylation levels between current-drinkers and non-drinkers.

In addition, the methylation levels of ACTB_CpG_2.3 and ACTB_CpG_7.8 showed inverse correlation with current drinking status in controls (Spearman rho = -0.191 and -0.242, respectively, *P* < 0.020 for both, Supplementary Table 6), but not with drinking status in stroke cases (Supplementary Table 6). Thus, we further analyzed the association between drinking and methylation levels of *ACTB*. As expected, current drinking status was associated with hypomethylation of ACTB_CpG_2.3 and ACTB_CpG_7.8 [for ACTB_CpG_2.3, median = 0.43 (IQR = 0.38–0.50) and 0.50 (IQR = 0.41–0.56) in current-drinkers and non-drinkers, respectively, and OR per + 10% methylation = 0.58, 95% CI: 0.37–0.91, *P* = 0.018 by logistic regression adjusting for age and sex; for ACTB_CpG_7.8, median = 0.33 (IQR = 0.29–0.42) and 0.37 (IQR = 0.33–0.44) in current-drinkers and non-drinkers, respectively, and OR per + 10% methylation = 0.62, 95% CI: 0.39–0.97, *P* = 0.038 by logistic regression adjusting for age and sex, Figures 5A,B and Supplementary Table 8].

The other CpG loci in the *ACTB* amplicon showed no correlation with age nor drinking (Supplementary Tables 6–8). No correlation was observed between the methylation levels of any of the 11 CpG sites in the *ACTB* amplicon and sex or smoking (data not shown).

### The Expression of *ACTB* in Peripheral Blood Leukocytes

Next, we sought to investigate whether the hypomethylation observed at the ACTB_CpG_14 site might influence the expression of *ACTB*. However, RNA materials were available only from 46 stroke cases. We thus analyzed the *ACTB* expression from the 46 stroke cases and another 48 age- and sex- matched healthy controls from the same prospective cohort study (Supplementary Table 9). The mean of the relative expression levels of *ACTB* in the leukocytes of stroke cases was 1.10-fold higher than in the controls (*ACTB* relative expression, mean ± SD of cases: 1.15 ± 0.36, mean ± SD of controls: 1.05 ± 0.38, Supplementary Figure 2). Presumably due to the limited sample size, we did not observe a significant difference in this comparison (*P* = 0.334).

## Discussion

In this nested case-control study, we have observed a relatively strong association between decreased methylation of ACTB_CpG_14 in peripheral blood DNA and onset-time-related increased risk of stroke. The prospective data showed pronounced evidence of differential methylation in peripheral blood and are detectable in the early phase before the clinical diagnosis of stroke. This hypomethylation of *ACTB* in blood became significant 1.5 years before the clinical indication of stroke, and was even more pronounced 1.32 years and 1 year preclinically.

Abnormal remodeling of vasculature, especially the resistance vessels, has been considered as an important risk factor for cardiovascular diseases (Qi et al., 2018). The RhoA and Rho kinase (ROCK) pathway regulates cell morphology by controlling cytoskeletal architecture and initiating vasoconstriction and vascular remodeling in hypertensive vessels (Chapados et al., 2006). As a downstream effector in the Rho/ROCK pathway, β-actin is a major component of the cytoskeleton and plays an important role in intercellular adhesion and contraction (Dugina et al., 2019). Karakozova et al. (2006) reported that the arginylation of β-actin *in vivo* could regulate actin cytoskeleton and cell motility, which may contribute to cardiovascular development and angiogenesis. Ji et al. (2007) demonstrated that the polymerization status of β-actin crucially regulated platelet nitric oxide synthase 3 activity in human platelets, thereby promoting nitric oxide formation, an important paracrine mediator of vascular function. These observations indicated critical roles of β-actin in vascular development and remodeling.

The *ACTB* gene, a well-known housekeeping gene, has been widely used to normalize the expression of genes in cell lines and in variant human tissue samples (Barr et al., 2010; Petrone et al., 2016; Raman et al., 2016). However, some studies showed that *ACTB* might not be an optimal reference gene for the normalization of gene expression in whole blood under the pressure of certain diseases such as stroke, which induces alterations in the cellular composition of the peripheral immune system (Stamova et al., 2009; Sieber et al., 2010). Additionally, our previous studies have shown that *ACTB* variants may confer genetic susceptibility to stroke and diabetic kidney disease (DKD) in a Chinese Han population (Li et al., 2019; Yang et al., 2020). However, little is yet known about the epigenetic impact of *ACTB* on the initiation and progress of stroke. In this study, we evaluated the association between *ACTB* methylation and the risk of stroke in a nested case-control study. To our knowledge, this is the first prospective study to report a significant association between blood-based *ACTB* hypomethylation and increased risk of stroke. The inverse association was more pronounced in stroke cases with shorter onset time, showing increasing alteration from control subjects. In addition, the three lower quartiles of ACTB_CpG_14 methylation levels were associated with an increased cumulative incidence of stroke. Based on these findings, we suggest that the methylation level of *ACTB* in blood DNA might be a preclinical marker in stroke and reflect a response to stroke in the years preceding clinical diagnosis, which is also supported by previous studies using pre-diagnostic blood samples (Xu et al., 2013, 2020). Nevertheless, only CpG_14 located in the fourth exon of the *ACTB* gene showed a significant correlation with stroke. It is possible that there might be specific methylation patterns in stroke, but it is also possible that the CpG_14 locus might contribute independently to the presence of stroke. Although we have observed a slightly increased expression of *ACTB* in the preclinical stroke cases, it was not significant due to the limited sample size. So far, no public DNA methylation database has ever reported the *ACTB* methylation in stroke. Knowing these limitations, we have just initiated another prospective cohort study with 20,000 subjects in Nanjing from January to March 2021. In the future, the association between *ACTB* methylation and risk of stroke, as well as its correlation with expression, will be further investigated in our new prospective cohort study. Meanwhile, we also call for validation by other prospective multi-center studies. In addition, our prospective data showed that differential methylation in the peripheral blood might exist before the clinical diagnosis of stroke. To know if the *ACTB* methylation is also associated with the early onset of stroke, a case-control design with large samples will be needed.

Differences of methylation signatures in peripheral blood might be influenced by the proportions of white blood cell subtypes, if the cell distribution itself differs by disease status. Extensive studies have indicated that stroke patients had a significant elevation in leukocyte and neutrophil counts in peripheral blood (Buck et al., 2008; Kriz and Lalancette-Hebert, 2009; Mo et al., 2013). In our study, the increase in leukocyte counts and neutrophil proportion was confirmed in the stroke cases. Nevertheless, after adjusting of cell proportions, ACTB_CpG_14 still showed a significant difference between stroke cases and controls.

Mounting evidence has disclosed the DNA methylation is related to age (Jung et al., 2017; Chen et al., 2019), and several environmental factors such as alcohol drinking, cigarette smoking and exercise (Philibert et al., 2012; Zhu et al., 2016). An age-related global DNA hypomethylation has been observed in rats (Pogribny et al., 2004), mice (Wilson et al., 1987), and human beings (Issa et al., 1994). In our study, we surprisedly only observed an inverse correlation between methylation levels of ACTB_CpG_4.5 and age in controls but not in cases, and consequently significantly lower ACTB_CpG_4.5 methylation in cases than controls in subjects < 65 years, but not in subjects ≥ 65 years. Additionally, we also found an inverse correlation between methylation levels of ACTB_CpG_2.3 and ACTB_CpG_7.8 and alcohol consumption in controls but not in cases, which is consistent with the fact drinkers have a higher risk of stroke (Kadlecova et al., 2015). It is unknown why the decreased *ACTB* methylation was only associated with aging and drinking in controls, and further validation is needed.

A particular strength of this study is the prospectively collected samples with careful uniform processing and storage, which minimized the possibility that the observed differences between stroke cases and controls might due to treatment or processing effects. However, we have to admit that our results on ACTB hypomethylation-related risk of stroke were based on a relatively small sample size, which power although could be compensated by the randomly selected samples from the whole prospective cohort.

## Conclusion

In summary, this study disclosed a significant correlation between preclinical altered *ACTB* methylation in blood and stroke, and thus suggested the potential of blood-based *ACTB* methylation for the early detection and even prevention of stroke. Further prospective studies with larger sample sizes and different stroke subtypes are needed to evaluate the potential value of *ACTB* methylation as a biomarker for stroke.

## Data Availability Statement

The original contributions presented in the study are included in the article/Supplementary Material, further inquiries can be directed to the corresponding author/s.

## Ethics Statement

This study was approved by the Ethics Committee of Nanjing Medical University. The patients/participants provided their written informed consent to participate in this study.

## Author Contributions

CL conducted the data collection, data analysis, and drafting the article. QY, ML, and YF collected the data. CS and RY performed the conception or design of the work, data interpretation, and critical revision of the article. All authors contributed to the article and approved the submitted version.

## Conflict of Interest

RY, CS, CL, and QY were inventors of a provisional patent application relating to the subject matter of this manuscript and therefore declare a potential conflict of interests. The remaining authors declare that the research was conducted in the absence of any commercial or financial relationships that could be construed as a potential conflict of interest.
