# Supplementary material for: ACTB Methylation in Blood as a Potential Marker for the Pre-clinical Detection of Stroke: A Prospective Nested Case-Control Study
Source: Front Neurosci. 2021 May 14;15:644943. doi: 10.3389/fnins.2021.644943 (PMC8160447; doi:10.3389/fnins.2021.644943)
Supplement: Supplementary file 1 [file Data_Sheet_1.doc]

**Supplementary** **Materials**

***Supplementary Table1. Methylation difference of ACTB between 147 controls and 139 stroke cases with onset time < 2 years***

| CpG sites | Controls | Stroke cases | Crude OR (95% CI) | *P -* value | OR (95% CI)* | *P -* value* |
| --- | --- | --- | --- | --- | --- | --- |
|  | median (IQR) | median (IQR) | per +10% methylation |  | per +10% methylation |  |
| ACTB_CpG_2.3 | 0.47 (0.40-0.54) | 0.47 (0.39-0.53) | 0.87 (0.69-­1.09) | 0.232 | 0.85 (0.68­-1.08) | 0.234 |
| ACTB_CpG_4.5 | 0.24 (0.18-0.30) | 0.24 (0.17-0.30) | 1.04 (0. 80-­1.35) | 0.782 | 0.95 (0.72­-1.27) | 0.737 |
| ACTB_CpG_6 | 0.43 (0.34-0.53) | 0.43 (0.31-0.50) | 0.92 (0.81-­1.06) | 0.241 | 0.91 (0.79­-1.05) | 0.195 |
| ACTB_CpG_7.8 | 0.36 (0.31-0.44) | 0.36 (0.29-0.43) | 0.86 (0.67-­1.11) | 0.253 | 0.88 (0.65­-1.14) | 0.289 |
| ACTB_CpG_9.10 | 0.32 (0.26-0.36) | 0.32 (0.26-0.36) | 0.98 (0.71-­1.35) | 0.898 | 0.85 (0.59­-1.22) | 0.369 |
| ACTB_CpG_11 | 0.61 (0.53-0.68) | 0.61 (0.49-0.71) | 1.01 (0.88-­1.16) | 0.869 | 0.98 (0.85­-1.13) | 0.745 |
| ACTB_CpG_12 | 0.24 (0.20-0.30) | 0.24 (0.18-0.31) | 0.94 (0.71-­1.25) | 0.670 | 0.92 (0.68­-1.25) | 0.589 |
| ACTB_CpG_14 | 0.51 (0.37-0.69) | 0.54 (0.39-0.66) | 0.99 (0.89-­1.11) | 0.873 | 0.93 (0.82­-1.06) | 0.264 |
| ACTB_CpG_15.16 | 0.50 (0.44-0.57) | 0.50 (0.43-0.57) | 1.01 (0.80-­1.29) | 0.911 | 0.91 (0.69-­1.20) | 0.523 |
| ACTB_CpG_17 | 0.29 (0.25-0.35) | 0.30 (0.25-0.36) | 1.06 (0.79-­1.43) | 0.683 | 0.99 (0.72­-1.36) | 0.944 |
| ACTB_CpG_18 | 0.30 (0.25-0.35) | 0.30 (0.24-0.35) | 0.96 (0.71-­1.29) | 0.769 | 0.88 (0.64­-1.21) | 0.418 |

***Logistic regression adjusting for BMI, smoking, drinking, hypertension, diabetes, TC, TG, HDL-C, LDL-C, leukocyte** **counts and** proportions **of neutrophil, lymphocyte and monocyte**.

**Supplementary Table 2. Methylation difference of *ACTB* between 147 controls and 91 stroke cases with onset time ≤ 1.5 years**

| CpG sites | Controls | Stroke cases | Crude OR (95% CI) | *P -* value | OR (95% CI)* | *P -* value* |
| --- | --- | --- | --- | --- | --- | --- |
|  | median (IQR) | median (IQR) | per +10% methylation |  | per +10% methylation |  |
| ACTB_CpG_2.3 | 0.47 (0.40-0.54) | 0.48 (0.38-0.54) | 0.85 (0.65-­1.10) | 0.207 | 0.84 (0.65­-­1.09) | 0.202 |
| ACTB_CpG_4.5 | 0.24 (0.18-0.30) | 0.22 (0.17-0.30) | 0.93 (0.68-­1.26) | 0.622 | 0.89 (0.65­-­­1.23) | 0.483 |
| ACTB_CpG_6 | 0.43 (0.34-0.53) | 0.44 (0.31-0.50) | 0.86 (0.73-­1.02) | 0.085 | 0.84 (0.70­­-­1.01) | 0.095 |
| ACTB_CpG_7.8 | 0.36 (0.31-0.44) | 0.36 (0.28-0.42) | 0.82 (0.61-­1.09) | 0.171 | 0.83 (0.59­­-­1.12) | 0.168 |
| ACTB_CpG_9.10 | 0.32 (0.26-0.36) | 0.31 (0.25-0.36) | 0.93 (0.64-­1.35) | 0.699 | 0.80 (0.53­-­­1.22) | 0.301 |
| ACTB_CpG_11 | 0.61 (0.53-0.68) | 0.62 (0.50-0.71) | 0.99 (0.85-­1.16) | 0.927 | 0.96 (0.81­-­­1.14) | 0.645 |
| ACTB_CpG_12 | 0.24 (0.20-0.30) | 0.24 (0.19-0.31) | 1.01 (0.73-­1.40) | 0.933 | 1.00 (0.70­-­­1.43) | 0.988 |
| ACTB_CpG_14 | 0.51 (0.37-0.69) | 0.46 (0.31-0.61) | 0.83 (0.73-­0.95) | **0.008** | 0.76 (0.65­­-­0.89) | **0.001** |
| ACTB_CpG_15.16 | 0.50 (0.44-0.57) | 0.50 (0.42-0.57) | 0.96 (0.73-­1.26) | 0.741 | 0.86 (0.63­-­­1.18) | 0.347 |
| ACTB_CpG_17 | 0.29 (0.25-0.35) | 0.28 (0.24-0.35) | 0.90 (0.64-­1.25) | 0.517 | 0.84 (0.58­-­­1.22) | 0.363 |
| ACTB_CpG_18 | 0.30 (0.25-0.35) | 0.30 (0.24-0.34) | 0.93 (0.66­-1.31) | 0.681 | 0.84 (0.59­­-­1.21) | 0.360 |

***Logistic regression adjusting for BMI, smoking, drinking, hypertension, diabetes, TC, TG, HDL-C, LDL-C, leukocyte** **counts and** proportions **of neutrophil, lymphocyte and monocyte**.

**Supplementary Table 3. Methylation difference of *ACTB* between 147 controls and 67 stroke cases with onset time ≤ 1.32 years**

| CpG sites | Controls | Stroke cases | Crude OR (95% CI) | *P -* value | OR (95% CI)* | *P -* value* |
| --- | --- | --- | --- | --- | --- | --- |
|  | median (IQR) | median (IQR) | per +10% methylation |  | per +10% methylation |  |
| ACTB_CpG_2.3 | 0.47 (0.40-0.54) | 0.48 (0.37-0.53) | 0.86 (0.65-1.14) | 0.290 | 0.78 (0.58-­1.07) | 0.126 |
| ACTB_CpG_4.5 | 0.24 (0.18-0.30) | 0.21 (0.17-0.28) | 0.88 (0.63-1.23) | 0.447 | 0.85 (0.6­0-1.22) | 0.380 |
| ACTB_CpG_6 | 0.43 (0.34-0.53) | 0.44 (0.32-0.49) | 0.86 (0.72-1.04) | 0.121 | 0.85 (0.70-­1.04) | 0.117 |
| ACTB_CpG_7.8 | 0.36 (0.31-0.44) | 0.35 (0.27-0.43) | 0.80 (0.58-1.09) | 0.157 | 0.74 (0.52­-1.05) | 0.096 |
| ACTB_CpG_9.10 | 0.32 (0.26-0.36) | 0.31 (0.25-0.35) | 0.87 (0.58-1.30) | 0.491 | 0.78 (0.49­-1.22) | 0.275 |
| ACTB_CpG_11 | 0.61 (0.53-0.68) | 0.63 (0.52-0.71) | 1.06 (0.88-1.27) | 0.548 | 1.01 (0.84­-1.23) | 0.895 |
| ACTB_CpG_12 | 0.24 (0.20-0.30) | 0.24 (0.19-0.32) | 1.06 (0.74-1.51) | 0.759 | 1.04 (0.71-­1.52) | 0.859 |
| ACTB_CpG_14 | 0.51 (0.37-0.69) | 0.39 (0.29-0.54) | 0.68 (0.56-0.81) | **2.10E-05** | 0.59 (0.47-­0.72) | **7.82E-07** |
| ACTB_CpG_15.16 | 0.50 (0.44-0.57) | 0.50 (0.42-0.56) | 0.94 (0.69-1.27) | 0.680 | 0.88 (0.62-­1.25) | 0.482 |
| ACTB_CpG_17 | 0.29 (0.25-0.35) | 0.28 (0.24-0.34) | 0.80 (0.55-1.17) | 0.254 | 0.76 (0.50-­1.15) | 0.197 |
| ACTB_CpG_18 | 0.30 (0.25-0.35) | 0.29 (0.24-0.34) | 0.88 (0.60-1.28) | 0.500 | 0.79 (0.52-­1.19) | 0.259 |

***Logistic regression adjusting for BMI, smoking, drinking, hypertension, diabetes, TC, TG, HDL-C, LDL-C, leukocyte** **counts and** proportions **of neutrophil, lymphocyte and monocyte**.

**Supplementary Table 4. Methylation difference of *ACTB* between 147 controls and 35 stroke cases with onset time ≤ 1 year**

| CpG sites | Controls | Stroke cases | Crude OR (95% CI) | *P -* value | OR (95% CI)* | *P -* value* |
| --- | --- | --- | --- | --- | --- | --- |
|  | median (IQR) | median (IQR) | per +10% methylation |  | per +10% methylation |  |
| ACTB_CpG_2.3 | 0.47 (0.40-0.54) | 0.45 (0.36-0.54) | 0.81 (0.56-1.17) | 0.264 | 0.80 (0.55-1.18) | 0.268 |
| ACTB_CpG_4.5 | 0.24 (0.18-0.30) | 0.21 (0.16-0.26) | 0.79 (0.51-1.22) | 0.284 | 0.78 (0.49­-1.22) | 0.267 |
| ACTB_CpG_6 | 0.43 (0.34-0.53) | 0.44 (0.30-0.50) | 0.85 (0.67-1.08) | 0.194 | 0.82 (0.63­-1.07) | 0.135 |
| ACTB_CpG_7.8 | 0.36 (0.31-0.44) | 0.34 (0.27-0.41) | 0.72 (0.48-1.11) | 0.134 | 0.71 (0.38-­1.12) | 0.161 |
| ACTB_CpG_9.10 | 0.32 (0.26-0.36) | 0.31 (0.25-0.35) | 0.84 (0.50-1.42) | 0.521 | 0.74 (0.41­-1.34) | 0.322 |
| ACTB_CpG_11 | 0.61 (0.53-0.68) | 0.62 (0.54-0.70) | 1.04 (0.82-1.31) | 0.751 | 1.01 (0.78-­1.31) | 0.931 |
| ACTB_CpG_12 | 0.24 (0.20-0.30) | 0.24 (0.19-0.34) | 1.20 (0.78-1.87) | 0.410 | 1.25 (0.76­-2.04) | 0.380 |
| ACTB_CpG_14 | 0.51 (0.37-0.69) | 0.37 (0.27-0.50) | 0.57 (0.43-0.75) | **5.90E-05** | 0.43 (0.3­0-0.61) | **3.00E-06** |
| ACTB_CpG_15.16 | 0.50 (0.44-0.57) | 0.47 (0.39-0.58) | 0.85 (0.57-1.27) | 0.427 | 0.72 (0.45-­1.15) | 0.166 |
| ACTB_CpG_17 | 0.29 (0.25-0.35) | 0.29 (0.24-0.35) | 0.89 (0.55-1.42) | 0.613 | 0.79 (0.47-­1.35) | 0.395 |
| ACTB_CpG_18 | 0.30 (0.25-0.35) | 0.29 (0.23-0.35) | 0.82 (0.51-1.34) | 0.430 | 0.75 (0.45­-1.27) | 0.284 |

***Logistic regression adjusting for BMI, smoking, drinking, hypertension, diabetes, TC, TG, HDL-C, LDL-C, leukocyte** **counts and** proportions **of neutrophil, lymphocyte and monocyte**.

**Supplementary Table 5. Correlation between *ACTB* methylation and onset time of stroke**

| CpG sites | Stroke onset time | | | | | | | | | | |
| --- | --- | --- | --- | --- | --- | --- | --- | --- | --- | --- | --- |
|  | ≤ 1 year | |  | ≤ 1.32 years | |  | ≤ 1.5 years | |  | < 2 years | |
|  | Spearman rho | *P* - value |  | Spearman rho | *P* - value |  | Spearman rho | *P* - value |  | Spearman rho | *P* - value |
| ACTB_CpG_2.3 | -0.089 | 0.610 |  | 0.035 | 0.777 |  | -0.004 | 0.971 |  | 0.001 | 0.989 |
| ACTB_CpG_4.5 | 0.120 | 0.493 |  | 0.094 | 0.447 |  | 0.107 | 0.315 |  | 0.155 | 0.070 |
| ACTB_CpG_6 | 0.033 | 0.852 |  | -0.062 | 0.620 |  | -0.012 | 0.913 |  | 0.025 | 0.771 |
| ACTB_CpG_7.8 | 0.079 | 0.652 |  | 0.030 | 0.812 |  | 0.088 | 0.408 |  | 0.088 | 0.302 |
| ACTB_CpG_9.10 | -0.140 | 0.424 |  | -0.070 | 0.573 |  | 0.038 | 0.724 |  | 0.051 | 0.553 |
| ACTB_CpG_11 | -0.071 | 0.685 |  | 0.018 | 0.886 |  | -0.141 | 0.184 |  | -0.075 | 0.384 |
| ACTB_CpG_12 | -0.042 | 0.813 |  | -0.087 | 0.484 |  | -0.086 | 0.422 |  | -0.104 | 0.226 |
| ACTB_CpG_14 | -0.081 | 0.645 |  | 0.283 | **0.021** |  | 0.534 | **5.89E-08** |  | 0.587 | **3.84E-14** |
| ACTB_CpG_15.16 | -0.097 | 0.581 |  | 0.044 | 0.726 |  | 0.048 | 0.653 |  | 0.092 | 0.283 |
| ACTB_CpG_17 | 0.170 | 0.330 |  | 0.009 | 0.945 |  | 0.081 | 0.448 |  | 0.203 | 0.017 |
| ACTB_CpG_18 | -0.098 | 0.577 |  | 0.024 | 0.848 |  | 0.072 | 0.497 |  | 0.026 | 0.766 |

**Supplementary Table 6. Correlation between *ACTB* methylation and age as well as drinking**

| CpG sites | Age (147 controls) | |  | Age (139 stroke cases) | |  | Current-drinking status (147 controls) | |  | Current-drinking status (139 stroke cases) | |
| --- | --- | --- | --- | --- | --- | --- | --- | --- | --- | --- | --- |
|  | Spearman rho | *P -* value |  | Spearman rho | *P -* value |  | Spearman rho | *P -* value |  | Spearman rho | *P -* value |
| ACTB_CpG_2.3 | 0.007 | 0.932 |  | 0.021 | 0.806 |  | -0.191 | **0.020** |  | 0.018 | 0.837 |
| ACTB_CpG_4.5 | -0.193 | **0.020** |  | 0.036 | 0.674 |  | -0.055 | 0.510 |  | -0.015 | 0.865 |
| ACTB_CpG_6 | -0.119 | 0.153 |  | -0.098 | 0.256 |  | -0.151 | 0.069 |  | -0.117 | 0.173 |
| ACTB_CpG_7.8 | 0.098 | 0.237 |  | 0.020 | 0.816 |  | -0.242 | **0.003** |  | -0.128 | 0.135 |
| ACTB_CpG_9.10 | -0.071 | 0.393 |  | 0.025 | 0.774 |  | -0.136 | 0.102 |  | -0.109 | 0.202 |
| ACTB_CpG_11 | -0.063 | 0.450 |  | -0.002 | 0.983 |  | 0.009 | 0.910 |  | 0.006 | 0.947 |
| ACTB_CpG_12 | -0.067 | 0.419 |  | -0.037 | 0.669 |  | -0.020 | 0.810 |  | -0.121 | 0.157 |
| ACTB_CpG_14 | 0.043 | 0.607 |  | 0.090 | 0.295 |  | -0.040 | 0.635 |  | 0.042 | 0.627 |
| ACTB_CpG_15.16 | 0.039 | 0.638 |  | 0.094 | 0.272 |  | -0.164 | 0.047 |  | -0.164 | 0.055 |
| ACTB_CpG_17 | 0.035 | 0.675 |  | -0.051 | 0.555 |  | -0.082 | 0.326 |  | -0.072 | 0.404 |
| ACTB_CpG_18 | -0.074 | 0.371 |  | 0.029 | 0.735 |  | -0.076 | 0.363 |  | -0.097 | 0.257 |

**Supplementary Table 7. Methylation difference of *ACTB* between 139 stroke cases and 147 controls stratiﬁed by age**

| CpG sites | Stratum | Controls | N | Stroke cases | N | Crude OR (95% CI) | *P -* value | OR (95% CI)* | *P -* value* |
| --- | --- | --- | --- | --- | --- | --- | --- | --- | --- |
|  |  | median (IQR) |  | median (IQR) |  | per +10% methylation |  | per +10% methylation |  |
| ACTB_CpG_2.3 | < 65 years | 0.45 (0.39-0.56) | 46 | 0.46 (0.38-0.52) | 50 | 0.84 (0.57-1.24) | 0.375 | 0.86 (0.54-­1.37) | 0.524 |
|  | ≥ 65 years | 0.49 (0.41-0.54) | 101 | 0.48 (0.40-0.54) | 88 | 0.90 (0.68-1.19) | 0.442 | 0.83 (0.62-­1.11) | 0.360 |
| ACTB_CpG_4.5 | < 65 years | 0.27 (0.19-0.32) | 46 | 0.24 (0.17-0.27) | 50 | 0.59 (0.36-0.97) | **0.039** | 0.56 (0.33­-0.98) | **0.042** |
|  | ≥ 65 years | 0.22 (0.18-0.29) | 99 | 0.25 (0.17-0.32) | 88 | 1.34 (0.97-1.86) | 0.079 | 1.30 (0.94-­1.81) | 0.131 |
|  |  |  |  |  |  |  |  |  |  |
| ACTB_CpG_6 | < 65 years | 0.45 (0.36-0.59) | 45 | 0.42 (0.32-0.49) | 49 | 0.84 (0.66-1.06) | 0.141 | 0.84 (0.66-­1.09) | 0.184 |
|  | ≥ 65 years | 0.42 (0.34-0.52) | 100 | 0.43 (0.31-0.53) | 87 | 0.96 (0.81-1.14) | 0.663 | 0.96 (0.79-­1.15) | 0.640 |
|  |  |  |  |  |  |  |  |  |  |
| ACTB_CpG_7.8 | < 65 years | 0.34 (0.29-0.43) | 46 | 0.35 (0.29-0.41) | 50 | 0.79 (0.50-1.25) | 0.321 | 0.79 (0.46­-1.34) | 0.373 |
|  | ≥ 65 years | 0.37 (0.32-0.44) | 101 | 0.37 (0.29-0.44) | 88 | 0.91 (0.67-1.23) | 0.532 | 0.89 (0.65­-1.22) | 0.480 |
|  |  |  |  |  |  |  |  |  |  |
| ACTB_CpG_9.10 | < 65 years | 0.32 (0.27-0.36) | 46 | 0.31 (0.25-0.35) | 50 | 0.75 (0.42-1.33) | 0.323 | 0.70 (0.36­-1.37) | 0.295 |
|  | ≥ 65 years | 0.31 (0.26-0.37) | 101 | 0.32 (0.26-0.37) | 88 | 1.12 (0.76-1.65) | 0.568 | 1.09 (0.72-1.66) | 0.691 |
|  |  |  |  |  |  |  |  |  |  |
| ACTB_CpG_11 | < 65 years | 0.61 (0.54-0.65) | 46 | 0.59 (0.49-0.69) | 50 | 0.93 (0.73-1.18) | 0.557 | 0.90 (0.69­-1.18) | 0.446 |
|  | ≥ 65 years | 0.61 (0.52-0.68) | 101 | 0.61 (0.50-0.71) | 88 | 1.05 (0.89-1.24) | 0.538 | 1.02 (0.85-­1.22) | 0.862 |
|  |  |  |  |  |  |  |  |  |  |
| ACTB_CpG_12 | < 65 years | 0.25 (0.20-0.31) | 46 | 0.24 (0.19-0.30) | 50 | 0.88 (0.54-1.45) | 0.614 | 0.86 (0.48­-1.56) | 0.617 |
|  | ≥ 65 years | 0.24 (0.20-0.29) | 101 | 0.24 (0.18-0.32) | 88 | 0.97 (0.69-1.36) | 0.846 | 0.91 (0.62­-1.33) | 0.630 |
|  |  |  |  |  |  |  |  |  |  |
| ACTB_CpG_14 | < 65 years | 0.50 (0.37-0.66) | 46 | 0.51 (0.33-0.63) | 50 | 0.93 (0.76-1.15) | 0.494 | 0.96 (0.75­-1.24) | 0.761 |
|  | ≥ 65 years | 0.51 (0.39-0.72) | 101 | 0.54 (0.40-0.71) | 88 | 1.03 (0.90-1.17) | 0.709 | 0.96 (0.82­-1.12) | 0.559 |
|  |  |  |  |  |  |  |  |  |  |
| ACTB_CpG_15.16 | < 65 years | 0.49 (0.43-0.57) | 46 | 0.48 (0.42-0.55) | 50 | 0.86 (0.56-1.30) | 0.461 | 0.81 (0.49­-1.33) | 0.407 |
|  | ≥ 65 years | 0.51 (0.44-0.56) | 101 | 0.50 (0.44-0.59) | 88 | 1.13 (0.83-1.52) | 0.441 | 0.94 (0.67­-1.33) | 0.743 |
|  |  |  |  |  |  |  |  |  |  |
| ACTB_CpG_17 | < 65 years | 0.29 (0.24-0.34) | 46 | 0.29 (0.24-0.36) | 50 | 1.04 (0.62-1.76) | 0.875 | 1.10 (0.63­-1.92) | 0.744 |
|  | ≥ 65 years | 0.29 (0.25-0.36) | 101 | 0.31 (0.25-0.36) | 88 | 1.09 (0.76-1.55) | 0.655 | 1.05 (0.70-1.56) | 0.663 |
|  |  |  |  |  |  |  |  |  |  |
| ACTB_CpG_18 | < 65 years | 0.31 (0.25-0.37) | 46 | 0.29 (0.24-0.33) | 50 | 0.76 (0.43-1.33) | 0.336 | 0.67 (0.36­-1.26) | 0.213 |
|  | ≥ 65 years | 0.30 (0.25-0.34) | 101 | 0.30 (0.25-0.36) | 88 | 1.05 (0.74-1.50) | 0.785 | 1.07 (0.72-1.53) | 0.864 |

***Logistic regression adjusting for BMI, smoking, drinking, hypertension, diabetes, TC, TG, HDL-C, LDL-C, leukocyte** **counts and** proportions **of neutrophil, lymphocyte and monocyte**.

**Supplementary Table 8. Methylation difference of *ACTB* between non-drinker and current drinkers**

| CpG sites | Group | Non-drinkers | N | Current-drinkers | N | Crude OR (95% CI) | *P -* value | OR (95% CI)* | *P -* value* |
| --- | --- | --- | --- | --- | --- | --- | --- | --- | --- |
|  |  | median (IQR) |  | median (IQR) |  | per +10% methylation |  | per +10% methylation |  |
| ACTB_CpG_2.3 | Control | 0.50 (0.41-0.56) | 100 | 0.43 (0.38-0.50) | 47 | 0.63 (0.42-0.94) | **0.023** | 0.58 (0.37-0.91) | **0.018** |
|  | Case | 0.47 (0.38-0.54) | 91 | 0.47 (0.40-0.52) | 47 | 0.98 (0.69-1.41) | 0.930 | 0.80 (0.53-1.21) | 0.289 |
|  |  |  |  |  |  |  |  |  |  |
| ACTB_CpG_4.5 | Control | 0.24 (0.19-0.29) | 98 | 0.22 (0.16-0.31) | 47 | 0.99 (0.64-1.51) | 0.945 | 0.92 (0.58-1.45) | 0.713 |
|  | Case | 0.24 (0.17-0.30) | 91 | 0.25 (0.17-0.31) | 47 | 0.99 (0.66-1.49) | 0.960 | 1.06 (0.66-1.70) | 0.813 |
|  |  |  |  |  |  |  |  |  |  |
| ACTB_CpG_6 | Control | 0.44 (0.35-0.55) | 99 | 0.38 (0.34-0.49) | 46 | 0.84 (0.66-1.06) | 0.144 | 0.78 (0.59-1.02) | 0.067 |
|  | Case | 0.44 (0.31-0.54) | 90 | 0.41 (0.32-0.48) | 46 | 0.88 (0.70-1.11) | 0.279 | 0.94 (0.73-1.22) | 0.634 |
|  |  |  |  |  |  |  |  |  |  |
| ACTB_CpG_7.8 | Control | 0.37 (0.33-0.44) | 100 | 0.33 (0.29-0.42) | 47 | 0.59 (0.38-0.91) | **0.018** | 0.62 (0.39-0.97) | **0.038** |
|  | Case | 0.36 (0.30-0.45) | 91 | 0.36 (0.27-0.40) | 47 | 0.71 (0.47-1.08) | 0.109 | 0.71 (0.44-1.14) | 0.154 |
|  |  |  |  |  |  |  |  |  |  |
| ACTB_CpG_9.10 | Control | 0.32 (0.26-0.37) | 100 | 0.30 (0.25-0.35) | 47 | 0.64 (0.39-1.04) | 0.074 | 0.58 (0.34-1.02) | 0.052 |
|  | Case | 0.32 (0.27-0.37) | 91 | 0.30 (0.25-0.36) | 47 | 0.79 (0.49-1.27) | 0.332 | 0.90 (0.53-1.54) | 0.706 |
|  |  |  |  |  |  |  |  |  |  |
| ACTB_CpG_11 | Control | 0.61 (0.54-0.68) | 100 | 0.61 (0.53-0.68) | 47 | 1.17 (0.93-1.48) | 0.178 | 1.14 (0.88-1.47) | 0.333 |
|  | Case | 0.60 (0.48-0.73) | 91 | 0.63 (0.50-0.69) | 47 | 0.96 (0.78-1.19) | 0.721 | 0.92 (0.73-1.16) | 0.485 |
|  |  |  |  |  |  |  |  |  |  |
| ACTB_CpG_12 | Control | 0.24 (0.20-0.30) | 100 | 0.24 (0.20-0.29) | 47 | 0.91 (0.56-1.46) | 0.681 | 0.92 (0.54-1.55) | 0.751 |
|  | Case | 0.24 (0.19-0.33) | 91 | 0.22 (0.17-0.27) | 47 | 0.73 (0.47-1.12) | 0.145 | 0.76 (0.48-1.20) | 0.24 |
|  |  |  |  |  |  |  |  |  |  |
| ACTB_CpG_14 | Control | 0.51 (0.37-0.68) | 100 | 0.51 (0.36-0.70) | 47 | 0.94 (0.79-1.13) | 0.522 | 0.89 (0.73-1.08) | 0.245 |
|  | Case | 0.53 (0.37-0.69) | 91 | 0.54 (0.39-0.64) | 47 | 1.00 (0.83-1.20) | 0.982 | 1.00 (0.82-1.22) | 0.968 |
|  |  |  |  |  |  |  |  |  |  |
| ACTB_CpG_15.16 | Control | 0.51 (0.46-0.57) | 100 | 0.49 (0.41-0.55) | 47 | 0.79 (0.53-1.18) | 0.242 | 0.76 (0.49-1.17) | 0.213 |
|  | Case | 0.50 (0.44-0.58) | 91 | 0.48 (0.40-0.54) | 47 | 0.83 (0.57-1.21) | 0.330 | 0.84 (0.55-1.26) | 0.393 |
|  |  |  |  |  |  |  |  |  |  |
| ACTB_CpG_17 | Control | 0.30 (0.25-0.36) | 100 | 0.29 (0.22-0.35) | 47 | 0.82 (0.52-1.29) | 0.394 | 0.78 (0.48-1.27) | 0.319 |
|  | Case | 0.30 (0.25-0.37) | 91 | 0.29 (0.24-0.36) | 47 | 0.83 (0.52-1.33) | 0.434 | 0.96 (0.57-1.59) | 0.859 |
|  |  |  |  |  |  |  |  |  |  |
| ACTB_CpG_18 | Control | 0.30 (0.25-0.37) | 100 | 0.30 (0.25-0.34) | 47 | 0.75 (0.46-1.23) | 0.250 | 0.70 (0.40-1.21) | 0.198 |
|  | Case | 0.31 (0.24-0.36) | 91 | 0.29 (0.23-0.32) | 47 | 0.74 (0.47-1.17) | 0.199 | 0.68 (0.40-1.15) | 0.145 |

***Logistic regression adjusting for age and sex**

| **Supplementary Table 9**. Description of the subjects for RNA analysis | | | | |
| --- | --- | --- | --- | --- |
| Characteristics | Controls | Stroke cases | t/χ2 | *P -*value |
|  | (n=48) | (n=46) |  |  |
| Age (year) | 67.98±8.41 | 67.46±8.50 | 0.302 | 0.764 |
| Gender |  |  |  |  |
| Male | 21 (43.8%) | 23 (47.9%) | 0.168 | 0.838 |
| Female | 27 (56.2%) | 25 (52.1%) |  |  |
| BMI (kg/m^2^) | 24.87±4.05 | 25.99±3.43 | 1.455 | 0.149 |
| SBP (mmHg) | 143.27±17.55 | 145.74±16.89 | 0.699 | 0.486 |
| DBP (mmHg) | 78.11±8.50 | 80.3±9.63 | 1.176 | 0.243 |
| Smoking status |  |  |  |  |
| Current smokers | 25 (52.1%) | 28 (58.3%) | 0.515 | 0.633 |
| Former smokers | 10 (20.8%) | 10 (20.8%) |  |  |
| Non-smokers | 13 (27.1%) | 10 (20.8%) |  |  |
| Drinking status |  |  |  |  |
| Yes | 35 (72.9%) | 40 (83.3%) | 1.524 | 0.324 |
| No | 13 (27.1%) | 8 (16.7%) |  |  |
| History of hypertension |  |  |  |  |
| Yes | 20 (41.7%) | 14 (29.2%) | 1.639 | 0.286 |
| No | 28 (58.3%) | 34 (70.8%) |  |  |
| History of diabetes |  |  |  |  |
| Yes | 43 (89.6%) | 33 (68.8%) | 6.316 | 0.022 |
| No | 5 (10.4%) | 15 (31.2%) |  |  |
| TC (mmol/L) | 4.90 ± 0.58 | 5.11±0.90 | 0.143 | 0.887 |
| TG (mmol/L) | 1.58±0.82 | 1.60±0.59 | 1.389 | 0.168 |
| HDL-C (mmol/L) | 1.50±0.36 | 1.40±0.35 | 1.335 | 0.185 |
| LDL-C (mmol/L) | 2.68±0.48 | 2.98±0.79 | 2.277 | 0.025 |
| Glucose (mmol/L) | 6.00±1.13 | 6.95±2.14 | 2.723 | 0.008 |
| Leukocytes (mil/mm3) | 6.29±1.27 | 6.66±1.66 | 1.237 | 0.219 |
| Neutrophils (%) | 55.65±8.02 | 56.60±8.13 | 0.580 | 0.563 |
| Lymphocytes (%) | 33.97±7.42 | 33.49±7.41 | 0.322 | 0.748 |
| Monocytes (%) | 7.53±1.64 | 7.27±1.83 | 0.724 | 0.471 |

**Supplementary Figures**

GGGACCTGACTGACTACCTCATGAAGATCCTCACCGAGCGCGGCTACAGCTTCACCACCACGGCCGAGCGGGAAATCGTGCGTGACATTAAGGAGAAGCTGTGCTACGTCGCCCTGGACTTCGAGCAAGAGATGGCCACGGCTGCTTCCAGCTCCTCCCTGGAGAAGAGCTACGAGCTGCCTGA**CG**GCCAGGTCATCACCATTGGCAATGAGCGGTTCCGCTGCCCTGAGGCACTCTTCCAGCCTTCCTTCCTGGGTGAGTGGAGACTGTCTCCCGGCTCTGCCTGACATGAGGGTTACCCCTCGGGGCTGTGCTGTGGAAGCTAAGTCCTGCCCTCATTTCCCTCTCAGGCATGGAGTCCTGTGGC

**Supplementary Figure 1.** Sequences of ACTB amplicon for MassARRAY methylation analysis (chr7: 5567503-5568411, build 37/hg 19, defined by the UCSC Genome Browser). The MassARRAY assay determined the methylation levels of sixteen CpGs and yielded eleven distinguishable peaks. Each of the 5 peaks contains two CpG sites including ACTB_ CpG_2 and ACTB_ CpG_3, ACTB_ CpG_4 and ACTB_ CpG_5, ACTB_ CpG_7 and ACTB_ CpG_8, ACTB_ CpG_9 and ACTB_ CpG_10, ACTB_ CpG_15 and ACTB_ CpG_16, whereas each of the other 6 peaks contains only one single CpG site. CpG sites that could be measured are depicted in blue. ACTB_ CpG_14 is underlined, in blue and bold.

**Supplementary Figure 2.** The expression of *ACTB* in peripheral blood leukocytes in controls and stroke cases. The dots represent the individual relative expression levels of *ACTB*. The *P*-value was calculated by t-test.
